# Methylation of the *KEAP1 *gene promoter region in human colorectal cancer

**DOI:** 10.1186/1471-2407-12-66

**Published:** 2012-02-13

**Authors:** Naoyuki Hanada, Takenori Takahata, Qiliang Zhou, Xulu Ye, Ruowen Sun, Jugoh Itoh, Atsushi Ishiguro, Hiroshi Kijima, Junsei Mimura, Ken Itoh, Shinsaku Fukuda, Yasuo Saijo

**Affiliations:** 1 Department of Medical Oncology, Hirosaki University Graduate School of Medicine, 5 Zaifu-cho, Hirosaki 036-8562, Japan; 2 Department of Gastroenterology and Hematology, Hirosaki University Graduate School of Medicine, 5 Zaifu-cho, Hirosaki 036-8562, Japan; 3 Department of Pathology and Bioscience, Hirosaki University Graduate School of Medicine, 5 Zaifu-cho, Hirosaki 036-8562, Japan; 4 Department of Stress Response Science, Hirosaki University Graduate School of Medicine, 5 Zaifu-cho, Hirosaki 036-8562, Japan; 5 Department of Rheumatology and Immunology, Shengjing Hospital of China Medical University, Shenyang, China

## Abstract

**Background:**

The Keap1-Nrf2 pathway has been reported to be impaired in several cancers. However, the status of Keap1-Nrf2 system in human colorectal cancer (CRC) has not been elucidated.

**Methods:**

We used colorectal cancer (CRC) cell lines and surgical specimens to investigate the methylation status of the *KEAP1 *promoter region as well as expression of Nrf2 and its downstream antioxidative stress genes, *NQO-1 *and *AKR1C1*.

**Results:**

DNA sequencing analysis indicated that all mutations detected were synonymous, with no amino acid substitutions. We showed by bisulfite genomic sequencing and methylation-specific PCR that eight of 10 CRC cell lines had hypermethylated CpG islands in the *KEAP1 *promoter region. HT29 cells with a hypermethylated *KEAP1 *promoter resulted in decreased mRNA and protein expression but unmethylated Colo320DM cells showed higher expression levels. In addition, treatment with the DNA methyltransferase inhibitor 5-Aza-dC combined with the histone deacetylase inhibitor trichostatin A (TSA) increased *KEAP1 *mRNA expression. These result suggested that methylation of the *KEAP1 *promoter regulates its mRNA level. Time course analysis with the Nrf2-antioxidant response element (ARE) pathway activator t-BHQ treatment showed a rapid response within 24 h. HT29 cells had higher basal expression levels of *NQO-1 *and *AKR1C1 *mRNA than Colo320DM cells. Aberrant promoter methylation of *KEAP1 *was detected in 53% of tumor tissues and 25% of normal mucosae from 40 surgical CRC specimens, indicating that cancerous tissue showed increased methylation of the *KEAP1 *promoter region, conferring a protective effect against cytotoxic anticancer drugs.

**Conclusion:**

Hypermethylation of the *KEAP1 *promoter region suppressed its mRNA expression and increased nuclear Nrf2 and downstream ARE gene expression in CRC cells and tissues.

## Background

Colorectal cancer (CRC) is the second leading cause of cancer-related deaths in most Western countries [1]. Over the past decade, molecular-targeted drugs have been applied in combination with cytotoxic agents. Consequently, the median overall survival for patients with advanced CRC has become longer than 24 months. Although the spectrum of therapeutic agents is becoming broader, many issues remain to be solved regarding cancer progression and acquisition of resistance to chemotherapy in CRC.

The Kelch-like ECH-associated protein 1 (Keap1) and nuclear factor-erythroid 2 -related factor 2 (Nrf2) pathway is one of the master regulators of cellular defense against oxidative and electrophilic stresses [2-4]. Nrf2 is a basic region-leucine zipper (bZip)-type transcription factor, which was identified as a binding protein of the β-globin gene locus [5,6]. Subsequently, Nrf2 was recognized to be a major transactivation factor for antioxidant response element (ARE)-dependent gene transcription [7]. The ARE is a *cis*-acting regulatory element of genes encoding phase II detoxification enzymes and antioxidant proteins, such as NAD(P)H quinone oxidoreductase-1 (NQO-1), glutathione S-transferases (GST), heme oxygenase-1 (HO-1), and aldo-keto reductase family 1 member C1 (AKR1C1). Keap1 is a negative regulator of Nrf2 and its main function is to serve as an adaptor for cullin3/ring box1 (Cul3/Rbx1) E3 ubiquitin ligase complex [8-12]. Under physiological conditions, Keap1 maintains a low basal level of Nrf2 by constantly targeting Nrf2 for ubiquitin-mediated protein degradation [13,14]. Once a cell is exposed to oxidative stress, Keap1 acts as a sensor and its cysteine residues are modified. This modification prevents rapid degradation of Nrf2, and the accumulated Nrf2 translocates into the nucleus, leading to active transcription of downstream cytoprotective genes.

The Keap1-Nrf2 signaling pathway is impaired in lung cancer, which is caused by mutations within functionally important domains of the *KEAP1 *or *NRF2 *gene [15-17]. Impaired Keap1 activity and somatic mutation of Nrf2 lead to full Nrf2 activation, and cancer cells may acquire a protective mechanism against the surrounding microenvironment, resulting in cancer cell proliferation, differentiation, and chemoresistance [15,17]. Similar *KEAP1 *mutations have been reported in patients with gall bladder cancer and in breast cancer cell lines [18,19].

Recently, Wang *et al*. reported that the promoter region of *KEAP1 *is aberrantly hypermethylated and *KEAP1 *mRNA expression levels are low in some lung cancer cell lines and lung cancer tissues [20]. Aberrant methylation of the *KEAP1 *promoter region was also reported in prostate cancer [21] and malignant glioma [22]. However, the methylation status of *KEAP1 *in CRC has not been elucidated.

As an impaired Keap1-Nrf2 system is induced by mutation or hypermethylation in several types of human cancer, we hypothesized that mutation or epigenetic changes of *KEAP1 *may decrease Keap1 expression and increase Nrf2 activity and transactivation of its downstream genes in CRC. In the present study, we investigated the methylation status of *KEAP1 *in 10 CRC cell lines and 40 surgically excised CRC tissue specimens. We found frequent hypermethylation of the *KEAP1 *gene promoter region in human CRC. In addition, the levels of Nrf2 target gene expression were upregulated in hypermethylated cells.

## Methods

### CRC cell lines and patient tissue samples

Human CRC cell lines were obtained from cell banks. The HT29 cell line was from American Type Culture Collection, while WiDr, LoVo, DLD-1, SW837, and Colo320DM cell lines were from the Human Science Research Resources Bank (Osaka, Japan). HCT15 and SW480 were from the Cell Resource Center for Biomedical Research Institute of Development, Aging, and Cancer, Tohoku University. TT1TKB and CW-2 were from RIKEN BioResource Center (Ibaraki, Japan). HT29, WiDr, LoVo, DLD-1, SW480, and SW837 were cultured in Dulbecco's Modified Eagle's Medium (DMEM) containing 10% heat-inactivated fetal bovine serum (FBS). HCT15, CW-2, and Colo320DM were cultured in RPMI1640 medium containing 10% FBS. Forty CRC tissues and adjacent normal colorectal tissue samples were collected with written informed consent at Hirosaki University Hospital. The tissues were immediately frozen and stored at -80°C after surgical resection. The study of CRC tissues samples was approved by the Ethics Committee of Hirosaki University School of Medicine.

### Cell treatment

HT29 cells were plated at 5 × 10^6 ^cells/10-cm dish 24 h prior to treatment. Cells were treated with 10 μM 5-aza-2'-deoxycytidine (5-Aza-dC) for 96 h to block CpG methylation, followed by treatment with 1 μM trichostatin A (TSA), a reversible inhibitor of histone deacetylase, for 24 h. To evaluate downstream gene expression of Nrf2, HT29 and Colo320DM cells were treated with 50 μM *tert*-butylhydroquinone (t-BHQ), a potent inducer of Nrf2-dependent gene expression, and cells were harvested at 2, 4, 8, 12, and 24 h after treatment. RNA was then extracted, and real-time reverse transcription-polymerase chain reaction (RT-PCR) was performed as described below to measure the expression of *NQO1 *and *AKR1C1*. The TaqMan Gene Expression Assay ID for the *NQO1 *mRNA is Hs00168547_m1, and that for *AKR1C1 *is Hs00413886_m1.

### DNA and RNA extraction and DNA sequencing of the *KEAP1 *gene

Genomic DNA was extracted from CRC cell lines using a QIAmp DNA Mini kit (Qiagen, Valencia, CA), and RNA was isolated using an RNeasy kit (Qiagen) according to the manufacturer's protocols. The DNA/RNA concentration and their quality were evaluated by measuring the ratio of optical density at 260/280 nm with NanoDrop (NanoDrop Technologies Wilmington, DE). For detection of *KEAP1 *mutation, DNA extracted from cell lines was amplified using AmpliTaq Gold^® ^Fast PCR Master Mix (Applied Biosystems, Carlsbad, CA). Direct sequencing was performed using the primer sets reported previously by Shibata *et al*. [18].

### Methylation-specific PCR (MSP) and bisulfite sequencing PCR (BSP) of the *KEAP1 *gene

**Figure 1:**
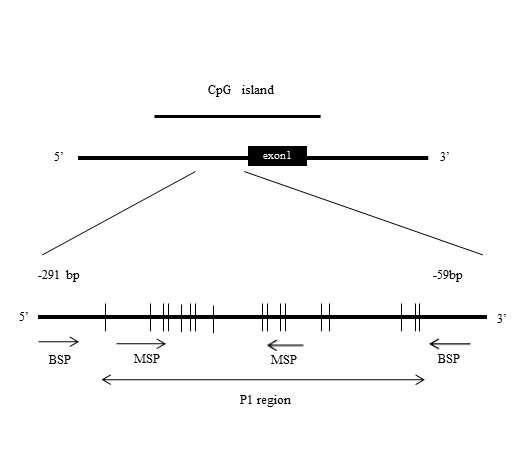
**Positions of methylation-specific PCR (MSP) and bisulfite sequencing PCR (BSP) primers**. BSP primers (-254 to -153) were designed to include the 12 CpG sites. MSP forward and reverse primers contained two and three CpG sites, respectively.

The primer sets of MSP and BSP used to target the CpG islands located in the putative promoter region of *KEAP1 *[20] are shown in Table 1 and Figure 1. These primer sets were designed using Methyl Primer Express Software v1.0 (Applied Biosystems), and PCR conditions for MSP and BSP are shown in Table 1. Aliquots of 2 μg of extracted DNA from CRC cell lines were converted using an Epitect Bisulfite kit (Qiagen) in accordance with the manufacturer's instructions. Direct DNA sequencing by dye terminator cycle sequencing was performed after bisulfite treatment using an ABI 310 Genetic analyzer (Applied Biosystems). PCR amplification with MSP primers was then performed using 10 μl of AmpliTaq Gold^® ^Fast PCR Master Mix and 20 ng of template DNA (the PCR conditions are shown in Table 1). CpG-methylated HeLa genomic DNA and 5-Aza-dC-treated Jurkat genomic DNA (New England Biolabs Japan, Tokyo, Japan) were used as controls for methylated and unmethylated sequence detection, respectively. MSP products were analyzed by 2% agarose gel electrophoresis, stained with ethidium bromide, and visualized with a UV transilluminator.

**Table 1:** PCR primers and thermal cycling conditions

Methods	Primers	Sequence
BSP		Forward: 5'-AAGAAAAGAAAAGAAAAGAAAATTTAG-3'
		Reverse: 5'-TTTAGTGAGGTAGATAATTTTTT-3'
PCR conditions		Initial denaturation at 95°C (10 min) and 35 cycles at 95°C (3 s), 52°C (3 s), 72°C (5 s), and a final extension at 72°C (10 s)

MSP	Methylation-	Forward: 5'-TAGATAATTTTTTTTAGATTTTGCGGTCG-3'
	Specific	Reverse: 5'-TCCTCGCGAAACTACGC-3'
		
PCR condition		Initial denaturation at 95°C (10 min) and annealing temperature decrement of 0.5°C every cycle (from 70°C to 66.5°C) followed by 32 cycles of 66°C (3 s), 72°C (5 s), and a final extension at 72°C (10 s)

MSP	Non-methylation	Forward: 5'-TAGATAATTTTTTTTAGATTTTGTGGTTG-3'
	-specific	Reverse: 5'-TCCTCACAAAACTACAC-3'
PCR condition		Initial denaturation at 95°C (10 min) and annealing temperature decrement of 0.5°C every cycle (from 64°C to 60.5°C) followed by 32 cycles of 60°C(3 s), 72°C (5 s), and a final extension at 72°C (10 s)

### Real-time RT-PCR

Expression of *KEAP1 *mRNA was measured by quantitative real-time PCR in triplicate using TaqMan Gene Expression Assays (Applied Biosystems) in the ABI PRISM 7000 sequence detection system (Applied Biosystems). Intact total RNA was extracted as described above. Reverse transcriptase reactions were performed on aliquots of 2 μg of total RNA using a High Capacity cDNA Reverse Transcription kit (Applied Biosystems) according to the manufacturer's protocol. The conditions for reverse transcription were 25°C (10 min), 37°C (120 min), and 85°C (5 min). The TaqMan Gene Expression Assay ID of the *KEAP1 *mRNA is Hs00202227_m1. Calculations were performed using the comparative C_T _method. GAPDH (Assay ID Hs99999905_m1) was used as an endogenous control gene for normalization of PCR for the amount of RNA added to the reverse transcription reactions. The mRNA levels are expressed as fold induction relative to the control. The conditions for real-time PCR were 50°C (2 min), 95°C (10 min), followed by 40 cycles of 95°C (15 s) and 60°C (1 min).

### Western blotting analysis

Whole-cell, cytoplasmic, and nuclear extracts from HT29 and Colo320DM cells were prepared using a Nuclear Extract kit (Active Motif, Tokyo, Japan) according to the manufacturer's instructions. The protein concentration was determined using a Pierce BCA Protein Assay Kit (Thermo Scientific, Waltham, MA). Whole-cell lysates containing 5 μg of protein from HT29 cells and 12.5 μg of protein from Colo320DM cells were loaded in each lane, run on a NuPAGE 4%-12% Bis-Tris gel (Invitrogen, Carlsbad, CA), and transferred onto PVDF iBlot Gel Transfer Stacks (Invitrogen). After blotting, membranes were blocked in Tris-buffered saline containing 0.05% Tween-20 (TBS-T) and 1% non-fat dried milk for 1 h. After blocking, membranes were probed overnight at 4°C with a rat monoclonal antibody against Keap1 (dilution 1:5,000; clone#144), a rabbit polyclonal antibody against Nrf2 (1:200; Santa Cruz, #sc-722), a mouse monoclonal antibody against NQO-1 (1:1,000; Santa Cruz Biotechnology, #sc-32793), and a mouse monoclonal antibody against AKR1C1 (1:1,000; ATGen,#ATGA0201). Membranes were washed four times (10 min per wash) with antibody dilution buffer and then incubated with goat anti-rabbit IgG (1:2,000; Santa Cruz Biotechnology) for 1 h at room temperature. A rabbit monoclonal antibody against β-actin (1:2,000; Cell Signaling Technologies, Danvers, MA) and a mouse monoclonal Antibody against histone H1 (1:500; Santa Cruz Biotechnology, #sc-8030) were used as controls. After extensive washing (4 × 10 min with TBS-T), antibody detection was performed with SuperSignal West Pico Chemiluminescent Substrate Kits (Pierce, Rockford, IL).

### Statistical analysis

Data are presented as the means ± standard deviation. Student's *t *test was used to assess the significance of three independent experiments. In all analyses, *P *< 0.05 was taken to indicate statistical significance.

## Results

### Genetic alteration of *KEAP1 *in CRC cell lines

As *KEAP1 *gene mutations have been reported in other types of human cancer, we sequenced all protein-coding exons in 10 CRC cell lines. We detected a C-to-T transition (G157G) in exon 2 of LoVo cells, a C-to-G transition (L470L) in exon 4 of LoVo, DLD-1, TT1TKB, HCT15, and CW-2 cells, and a C-to-T transition (Y537Y) in exon 5 of CW-2 cells. All mutations were single-nucleotide polymorphisms and had been reported previously. No missense or nonsense mutations were observed.

### Analysis of the methylation status of the *KEAP1 *promoter region in 10 CRC cell lines

**Figure 2:**
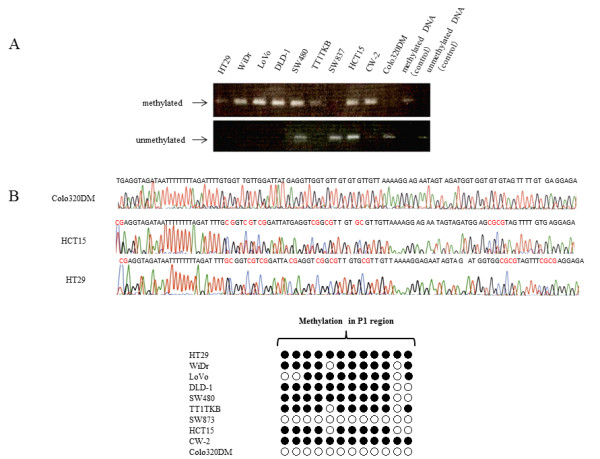
**Analysis of *KEAP1 *promoter region methylation status in CRC cell lines**. (**A**) Results of MSP in 10 CRC cell lines. (**B**) Results of BSP. Representative methylation analysis of CpG islands at the promoter region of the *KEAP1 *gene (upper). Methylation is indicated in red. Methylation status of each of the 12 CpG sites in 10 CRC cell lines (lower). Black circles represent methylated CpGs. White circles represent unmethylated CpGs.

The *KEAP1 *promoter region was hypermethylated in lung cancer cell lines and lung cancer tissues, as reported previously by Wang *et al*. [20]. They reported that the P1 region, including 12 CpGs (-291 to -89), was heavily hypermethylated in the CpG islands around the transcriptional initiation site of *KEAP1*. Therefore, we investigated the methylation status of the P1 region in *KEAP1 *using MSP and BSP primers designed as shown in Figure 1. MSP analysis indicated that the P1 region was hypermethylated in HT29, WiDr, LoVo, DLD-1, SW480, TT1TKB, HCT15, and CW-2 cells, but not in SW837 or Colo320DM (Figure 2A). Furthermore, we determined the methylation status of each of the 12 CpG dinucleotide sites in the P1 region by BSP. As shown in Figure 2B, most of CpG sites were methylated in HT29, WiDr, LoVo, DLD-1, SW480, TT1TKB, HCT15, and CW-2, but not in SW837 or Colo320DM. Representative results of methylation analysis of CpG islands in the promoter region of the *KEAP1 *gene are shown in Figure 2B. All cytosines in the P1 region were converted to thymidine in Colo320DM cells, although in HT29 cells the 5'-methylcytosines of CpG sites remained as cytosines. In contrast, both cytosines and thymidines in the 5'-methylcytosines of CpG sites were observed in HCT15 cells. Aberrant hypermethylation in the *KEAP1 *promoter region was frequently observed in human CRC cell lines.

### Association between *KEAP1 *methylation and *KEAP1 *mRNA expression

**Figure 3:**
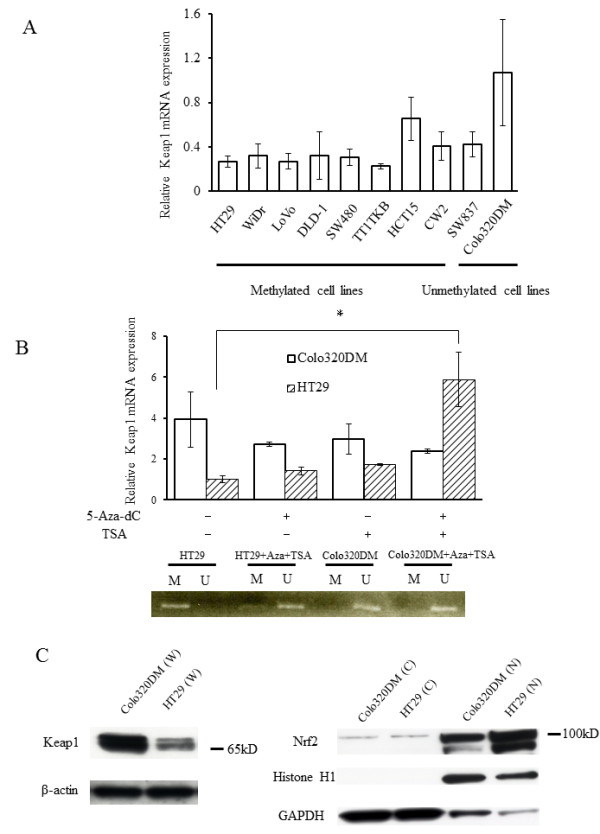
***KEAP1 *and *Nrf2 *expression**. (**A**) *KEAP1 *mRNA expression in 10 CRC cell lines was evaluated by real-time PCR. The expression level in Colo320DM cells was arbitrarily designated as 1. Columns, mean (*n *= 3); bars, standard deviation (SD). (**B**, upper) *KEAP1 *mRNA levels in HT29 cells (methylated) and Colo320DM cells (unmethylated) were analyzed by real-time PCR after treatment with 5-Aza-dC, TSA, and 5-Aza-dC + TSA. The expression level in HT29 cells was arbitrarily designated as 1. Columns, mean (*n *= 3); bars, SD. *P < 0.05. (**B**, lower) MSP analysis of in HT29 cells and Colo320DM treated with 5-Aza-dC + TSA. M, methylation-specific primer; U, non-methylation-specific primer. (**C**) Western blot analysis of Keap1 and Nrf2 in methylated and unmethylated colon cancer cells. Whole-cell extracts (W), cytosolic extracts (C), and nuclear extracts (N) were prepared from Colo320DM and HT29 cells. Extracts were stained with antibody to Keap1 or Nrf2A. β-Actin, histone H1, and GAPDH antibodies were used as loading controls for whole-cell, cytosolic, and nuclear fractions, respectively.

To examine the effects of *KEAP1 *methylation on its mRNA expression level, we performed real-time RT-PCR of *KEAP1 *mRNA as shown in Figure 3A. Cell lines with methylated *KEAP1 *(HT29, WiDr, LoVo, DLD-1, SW480, TT1TKB, HCT15, and CW-2) exhibited lower levels of *KEAP1 *mRNA expression compared with the unmethylated cell lines SW837 and Colo320DM.

To determine whether expression of *KEAP1 *mRNA is epigenetically downregulated, expression of *KEAP1 *mRNA was measured after treatment with the demethylating agent 5-Aza-dC at 10 μM for 4 days and/or the reversible histone deacetylase inhibitor TSA at 1 μM for 24 h in HT29 cells. The expression of *KEAP1 *mRNA was markedly increased after 5-Aza-dC and TSA treatment in the methylated cell line HT29, but no changes were observed in *KEAP1 *mRNA expression level in the unmethylated cell line Colo320DM (Figure 3B). MSP analysis showed that methylation of the *keap1 *promoter in HT29 cells was reversed after 5-Aza-dC and TSA treatment (Figure 3B). These observations suggest that epigenetic alterations regulate Keap1 expression in CRC cell lines.

### Protein levels of *Keap1 *and Nrf2

**Figure 4:**
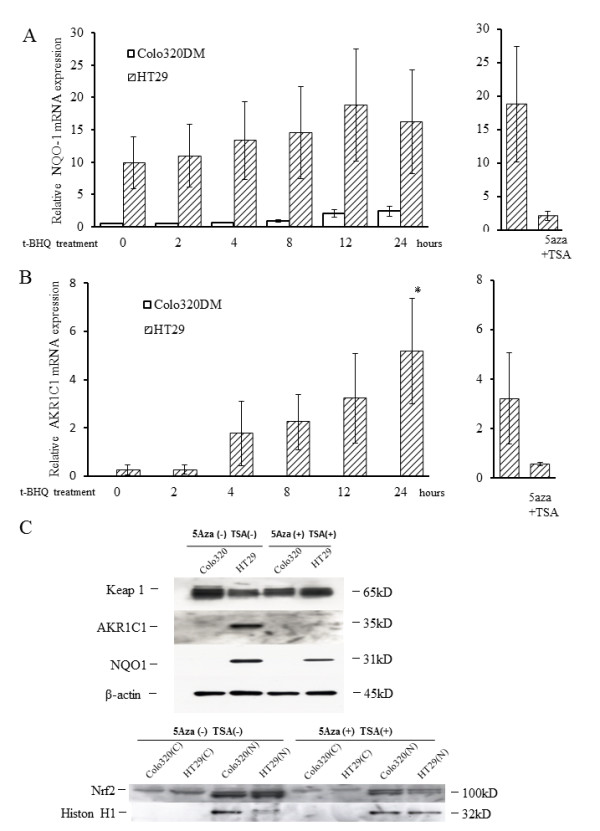
**Expression of the *Nrf2 *target genes *NQO-*1 and *AKR1C1 *after t-BHQ treatment**. Real-time PCR analysis of the *Nrf2 *target genes *NQO-1 *(**A**, left) and *AKRC1 *(**B**, left) in HT29 cells (methylated) and Colo320DM cells (unmethylated). Cells were treated with the Keap1 stimulator t-BHQ for 24 h. Columns, mean (*n *= 3); bars, SD. *P < 0.05. Real-time PCR analysis of *NQO-1 *(**A**, right) and *AKRC1 *(**B**, right) in HT29 cells treated with 5-Aza-dC + TSA and then t-BHQ (for 12 h). Keap1 protein levels (**C**, upper), protein expression of downstream ARE genes (**C**, upper), and changes in the subcellular distribution of Nrf2 protein (**C**, lower) after 5-Aza-dC and TSA treatment. Cells were treated with 5-Aza-dC and TSA. Expression levels of each protein were determined by Western blotting. β-actin and histone H1 were used as loading controls.

To further examine whether Keap1 protein levels are different between methylated and unmethylated cells, we performed Western blotting analysis. The Keap1 protein level was reduced in HT29 cells, compared with that in Colo320DM cells, as shown in Figure 3C (left). Keap1 protein expression in the methylated cell line HT29 was reversed by treatment with 5-Aza-dC and TSA, but was unchanged in the unmethylated cell line Colo320DM (Figure 4C), mirroring similar changes in KEAP1 mRNA expression. In addition, Nrf2 protein clearly accumulated in the nuclear fraction of HT29 cells, as compared to its level in Colo320DM, whereas Nrf2 protein levels in cytoplasmic fractions were equivalent in these two cell lines (Figure 3C, right). Nrf2 protein accumulation in HT29 cells was reduced by demethylation (Figure 4C).

### *NQO1 *and *AKR1C1 *mRNA and protein levels

We measured *NQO1 *and *AKR1C1 *mRNA levels at different time points after treatment with t-BHQ, an activator of the Nrf2-ARE pathway, at a concentration of100 μM. *NQO1 *and *AKR1C1 *expression levels were higher in the methylated cell line HT29 than in the unmethylated cell line Colo320DM without stimulation (Figures 4A and 4B left). Furthermore, t-BHQ treatment significantly increased *NQO-1 *and *AKR1C1 *mRNA levels in HT29 cells. *AKR1C1 *mRNA was below the limit of detection both at baseline and after stimulation in Colo320DM cells. The expression of *NQO1 *and *AKR1C1 *mRNA in the methylated cell line HT29 was reversed after treatment with 5-Aza-dC and TSA (Figures 4A and 4B (right)). NQO-1 and AKR1C1 proteins were overexpressed in methylated HT 29 cells, but their levels were reduced after demethylation (Figure 4C).

### Detection of *KEAP1 *methylation using MSP in surgical samples and association between methylation status and clinicopathological features in CRC

**Figure 5:**
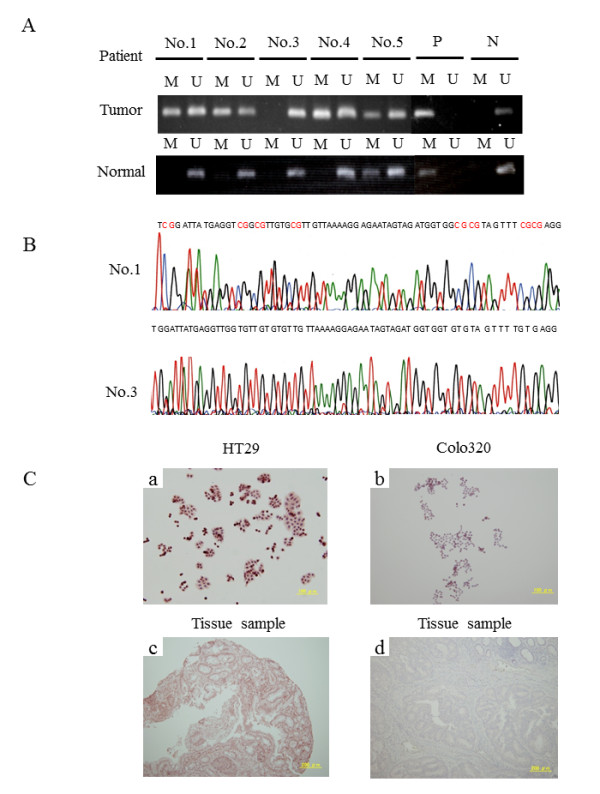
**Methylation of the *KEAP1 *promoter in CRC tissue samples**. (**A**) MSP for the *KEAP1 *promoter was performed using bisulfite-modified DNA from 40 CRC tissues and adjacent normal colorectal tissues. MSP results from 10 patients are shown. M: MSP of methylation-specific primers; U: MSP of non-methylation-specific primers; P: positive methylated DNA control; N: negative unmethylated DNA control. (**B**) Representative results of MSP sequence analysis of tumor tissues. The methylation status of the *KEAP1 *promoter region in patient tumor tissues was determined using methylation-specific primers (upper), and non-methylation-specific primers (lower). Methylation is indicated in red. (**C**) Expression and subcellular localization of Nrf2. Nrf2 was more highly expressed in HT29 cells (**a**) and a methylated tissue sample (**c**) compared with Colo320 cells (**b**) and a non-methylated tissue sample (**d**). Expression and localization of Nrf2 was studied using an anti-human Nrf2 antibody.

The methylation status of each sample was confirmed by MSP and BSP. Representative MSP products for *KEAP1 *in tumor tissues and normal tissues are shown in Figure 5A. Representative results of MSP sequence analysis of tumor tissues are presented in Figure 5B. Aberrant promoter methylation of *KEAP1 *was detected in 21/40 (53%) tumor tissues and 10/40 (25%) normal mucosal specimens (Table 2). Compared with normal mucosa, the methylation of *KEAP1 *was more prominent in tumor tissues (*P *= 0.001). We performed statistical analyses to determine whether the *KEAP1 *methylation status of colorectal tumor samples is associated with the clinicopathological features of CRC patients. In the tumor tissues, methylation of *KEAP1 *was not associated with any clinicopathological features, such as primary site location, differentiation, gender, Duke's stage, clinical stage, age, lymph node metastasis, and serum concentration of carcinoembryonic antigen (CEA) (data not shown). Additionally, we analyzed methylated HT29 cells and tumor samples by immunohistochemistry using an anti-human Nrf2 antibody. As shown in Figure 5C, strong expression of Nrf2 protein was detected in the nuclei of HT29 cells and in a methylated tissue sample. This observation indicates that promoter methylation of the *KEAP1 *gene enables Nrf2 to translocate from the cytoplasm to the nucleus.

**Table 2:** *KEAP1 *promoter methylation frequency in colorectal cancer and adjacent normal mucosa

		Methylation status	
		
Variable	N	Present	Absent
Tumor tissue	40	21 (53%)	19 (47%)
Adjacent normal mucosa	40	10 (25%)	30 (75%)

			*P *< 0.05

## Discussion and conclusions

We found frequent hypermethylation of the *KEAP1 *promoter region in human CRC cell lines. This hypermethylation of *KEAP1 *resulted in reductions in *KEAP1 *mRNA and protein expression, upregulation of Nrf2 activity, and thus overexpression of downstream genes, such as *NQO-1 *and *AKR1C1*. We also observed aberrant methylation of *KEAP1 *in human CRC tissues. This is the first report discussing activation of Keap1/Nrf2 signaling by *KEAP1 *hypermethylation in CRC.

Loss of Keap1 function has been reported associated with *KEAP1 *gene mutations in tumor tissue samples from lung, gall bladder, breast, and prostate cancer [15,18,19,21]. We found only synonymous mutations consisting of a C-to-T transition with G157G in exon 2, a T-to-C transition of L471L in the DGR4 domain, and a C-to-T transition with Y537Y in the DGR5 domain in CRC cell lines. However, these mutations were single-nucleotide polymorphisms. Frequent *KEAP1 *gene mutations were reported in human non-small cell lung cancer (NSCLC) [15]. All mutations were within highly conserved amino acid residues located in the Kelch or intervening region domain of the Keap1 protein, suggesting that these mutations were likely to abolish Keap1 repressor activity against Nrf2. In addition, C23Y mutation in the N-terminal domain of Keap1 has been reported to have impaired ability to repress Nrf2 activity due to its inability to stimulate the ubiquitylation and degradation of Nrf2 in breast cancer [19]. A C-to-T transition with T314M and a T-to-C transition with Y255H were detected in six prostatic cancer cell lines [21]. Shibata *et al*. also reported mutations of *KEAP1 *in biliary tract cancer tissue [18]. These changes are in the central intervening region of Keap1 and alter highly conserved amino acids.

Another mechanism of impaired Keap1 activity is hypermethylation of *KEAP1*. We found that 8 of 10 CRC cell lines had methylated CpG islands in the promoter region of the *KEAP1 *gene where methylation was found in other types of cancer [20,22,23]. Hypermethylation of *KEAP1 *resulted in decreased mRNA expression, which was confirmed by the increase in *KEAP1 *mRNA expression by combined treatment with the DNA methyltransferase inhibitor 5-Aza-dC and the histone deacetylase inhibitor TSA (Figure 4C). Hypermethylation of *KEAP1 *caused final stimulation of Nrf2 target genes. However, the reason for the expression of *KEAP1 *mRNA being lower in unmethylated SW837 cells than in methylated HCT15 cells is unknown. Wang *et al*. investigated three lung cancer cell lines and five tumor samples, and found frequent hypermethylation of the CpG islands in the promoter region of *KEAP1 *and reduced levels of *KEAP1 *mRNA expression. In contrast, a normal bronchial cell line had clearly less methylation of the *KEAP1 *promoter region and elevated mRNA expression [20]. Hypermethylation of *KEAP1 *found in prostate cancer also stimulated the Nrf2 signal [21].

Biological effects of constitutive Nrf2 activation by Keap1 dysfunction due to mutations or low-level expression by hypermethylation have been reported previously [18,23,24]. Constitutive expression of the cytoprotective gene by Nrf2 activation in lung cancer cells led to chemotherapy resistance [23]. Nrf2 activation also stimulated growth of lung cancer cells. Nrf2 activation by *KEAP1 *mutation or hypermethylation of promoter CpG islands causes radioresistance and promotes tumor growth in prostatic cancer [21]. In the present study, we observed accumulation of Nrf2 protein in the nuclei in methylated HT29 cells, and overexpression of phase II detoxifying enzymes NQO-1 and AKR1C1 both at baseline and after t-BHQ stimulation. These reports indicate that KEAP1 functions as a tumor suppressor gene in human tumors. Although we did not evaluate the biological effects of activated Nrf2, we assume that CRC cells with *KEAP1 *gene hypermethylation may be resistant to chemotherapeutic agents and show upregulated cell growth, as reported in other types of cancer.

There have been only two previous reports regarding Keap1/Nrf2 l in CRC cells [24,25]. Activation of the Keap1/Nrf2 signaling pathway mediates protective responses to mitigate nitric oxide (NO)-induced damage and may contribute to the resistance of CRC cells to NO-induced cytotoxicity [24]. Arlt *et al*. reported that Nrf2 activity is elevated in colon cancer, accounting for overexpression of the proteasome subunit proteins and thus for increased proteasome activity [25]. Conversely, small interfering RNA-mediated Nrf2 knockdown decreased their expression and reduced proteasome activity, thus indicating that Nrf2 is related to colorectal carcinogenesis. This Nrf2 activation may be due to the low level of Keap1 expression due to hypermethylation, as found in the present study.

Biological effects that activate Nrf2 signaling prompted us to study the relationship between the status of Keap1/Nrf2 signaling and clinicopathological features of the tumors. Type II endometrial cancer, which is mostly malignant and is associated with a poor prognosis among gynecological malignancies, shows elevated Nrf2 protein expression, whereas benign tumors and type I endometrial cancer do not [26]. On immunohistochemical analysis of human NSCLC, increased Nrf2 expression and low or absent Keap1 expression were associated with worse survival [27]. In contrast, the prognosis of malignant glioma was better among patients with than among those without a methylated *KEAP1 *promoter region [22]. Although we did not investigate the prognosis of patients with CRC, further studies are needed to understand the role of Keap1/Nrf2 signaling in human CRC.

In conclusion, the results of the present study revealed hypermethylation of the *KEAP1 *promoter region in human CRC, leading to downregulation of *KEAP1 *mRNA expression, thus activating Nrf2 and expression of its downstream target genes. Cancerous tissues exhibited more frequent methylation of *KEAP1 *than normal tissue in surgically resected CRC specimens.

## Competing interests

The authors declare that they have no competing interests.

## Authors' contributions

HN, TT, ZQ, YX, SR, and MJ performed experiments and summarized the data. IJ, IA, IK, FS, and SY designed the experiments. HN, TT, and SY wrote the paper; all authors have read and approved the final manuscript.

## Pre-publication history

The pre-publication history for this paper can be accessed here:

http://www.biomedcentral.com/1471-2407/12/66/prepub
